# *N*-{4-[4-(4-Fluoro­phen­yl)-1-(2-methoxy­ethyl)-2-methyl­sulfanyl-1*H*-imidazol-5-yl]-2-pyrid­yl}-2-methyl-3-phenyl­propionamide

**DOI:** 10.1107/S1600536809048661

**Published:** 2009-11-21

**Authors:** Katharina Ziegler, Dieter Schollmeyer, Stefan Laufer

**Affiliations:** a Institute of Pharmacy, Department of Pharmaceutical and Medicinal Chemistry, Eberhard-Karls-University Tübingen, Auf der Morgenstelle 8, 72076 Tübingen, Germany; b Department of Organic Chemistry, Johannes Gutenberg-University Mainz, Duesbergweg 10-14, D-55099 Mainz, Germany

## Abstract

In the crystal structure of the title compound, C_28_H_29_FN_4_O_2_S, the imidazole ring makes dihedral angles of 11.85 (7), 73.33 (7) and 22.83 (8)° with the 4-fluoro­phenyl, pyridine and phenyl rings, respectively. The 4-fluoro­phenyl ring makes dihedral angles of 77.91 (7) and 26.93 (8)° with the pyridine and phenyl rings, respectively. The phenyl and pyridine rings are nearly perpendicular, making a dihedral angle of 86.47 (9)°. The crystal packing shows an inter­molecular N—H⋯O hydrogen-bonding inter­action between the N—H and carbonyl groups of the amide functions.

## Related literature

For related compounds and their biological activity, see: Laufer *et al.* (2004 ▶). For the biological activity of the title compound, see: Ziegler *et al.* (2009 ▶).
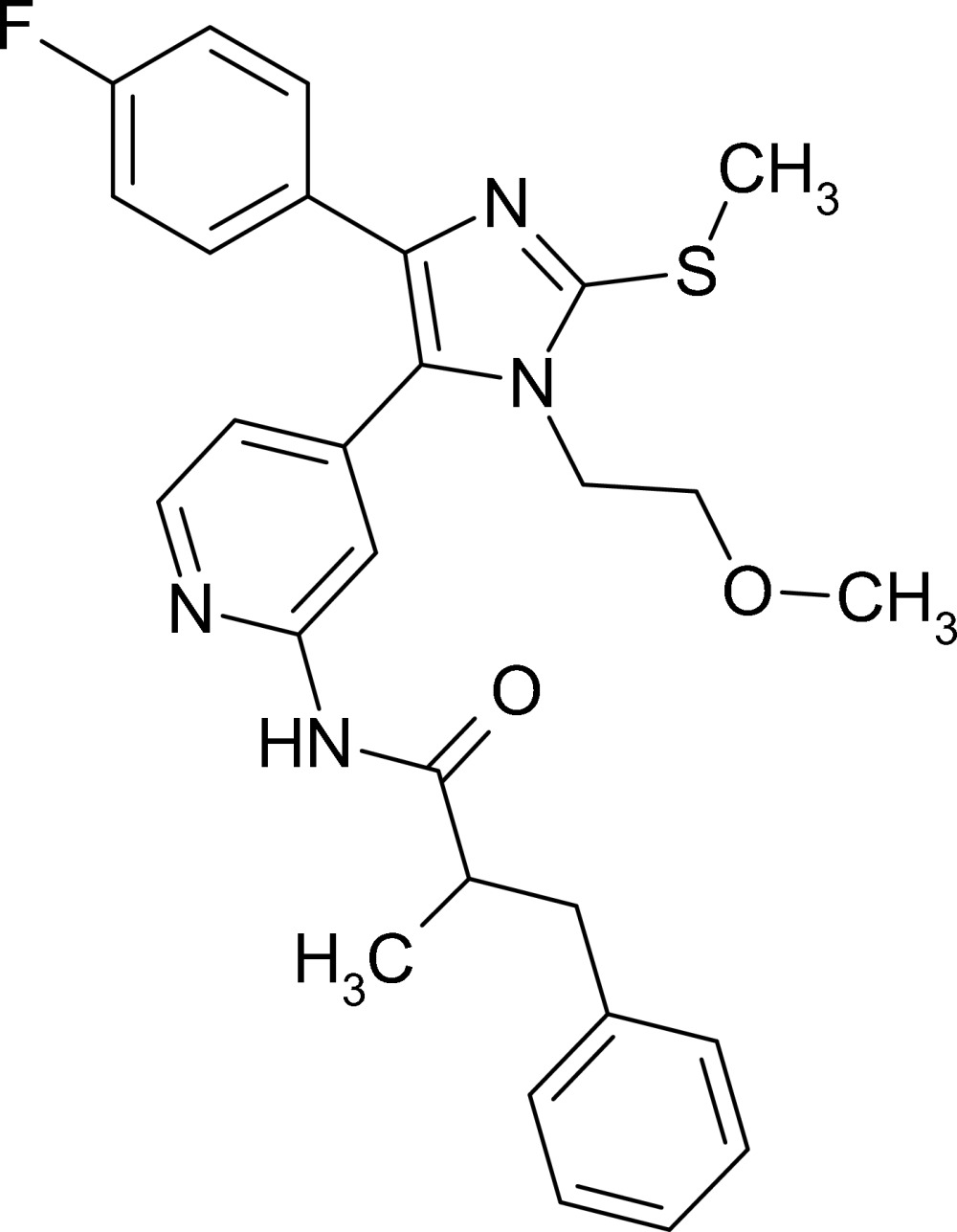


## Experimental

### 

#### Crystal data

C_28_H_29_FN_4_O_2_S
*M* *_r_* = 504.61
Monoclinic, 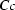

*a* = 10.6254 (5) Å
*b* = 28.542 (1) Å
*c* = 9.8380 (4) Å
β = 117.953 (1)°
*V* = 2635.5 (2) Å^3^
*Z* = 4
Mo *K*α radiation
μ = 0.16 mm^−1^
*T* = 173 K
0.50 × 0.50 × 0.20 mm

#### Data collection

Bruker SMART with APEXII CCD diffractometer
Absorption correction: multi-scan (*SADABS*; Bruker, 2006 ▶) *T*_min_ = 0.685, *T*_max_ = 0.746
21744 measured reflections
6183 independent reflections
6019 reflections with *I* > 2σ(*I*)
*R*_int_ = 0.020

#### Refinement

*R*[*F* ^2^ > 2σ(*F* ^2^)] = 0.029
*wR*(*F* ^2^) = 0.078
*S* = 1.03
6183 reflections
328 parameters
2 restraints
H-atom parameters constrained
Δρ_max_ = 0.27 e Å^−3^
Δρ_min_ = −0.17 e Å^−3^
Absolute structure: Flack (1983 ▶), 3058 Friedel pairs
Flack parameter: 0.13 (4)

### 

Data collection: *APEX2* (Bruker, 2006 ▶); cell refinement: *SAINT* (Bruker, 2006 ▶); data reduction: *SAINT*; program(s) used to solve structure: *SIR97* (Altomare *et al.*, 1999 ▶); program(s) used to refine structure: *SHELXL97* (Sheldrick, 2008 ▶); molecular graphics: *PLATON* (Spek, 2009 ▶); software used to prepare material for publication: *PLATON*.

## Supplementary Material

Crystal structure: contains datablocks I, global. DOI: 10.1107/S1600536809048661/im2161sup1.cif

Structure factors: contains datablocks I. DOI: 10.1107/S1600536809048661/im2161Isup2.hkl

Additional supplementary materials:  crystallographic information; 3D view; checkCIF report

## Figures and Tables

**Table 1:** Hydrogen-bond geometry (Å, °)

*D*—H⋯*A*	*D*—H	H⋯*A*	*D*⋯*A*	*D*—H⋯*A*
N7—H7⋯O9^i^	0.82	2.21	3.025 (1)	173

Symmetry code: (i) 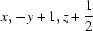.

**Fig. 1.**
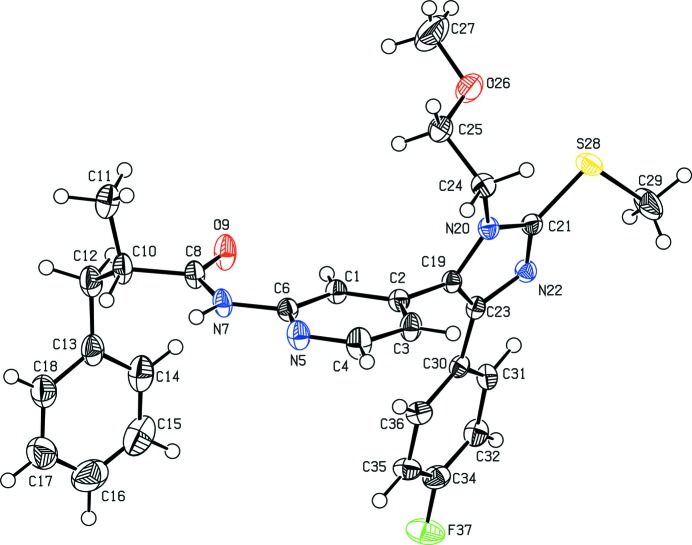
View of compound I. Displacement ellipsoids are drawn at the 50% probability level. H atoms are depicted as circles of arbitrary size.

**Fig. 2.**
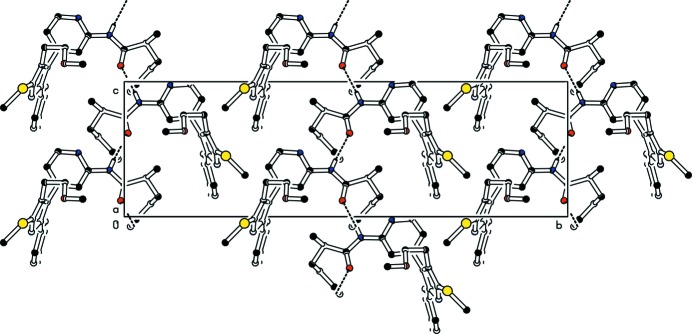
Part of the crystal packing of compound I. The hydrogen bond is shown with dashed lines. View along a axis.

## supplementary crystallographic information

### Comment

N1 substituted 2-alkylsulfanyl-4-(4-fluorophenyl)-5-(pyridin-4-yl)-imidazoles are known to be potent p38 mitogen-activated protein (MAP) kinase inhibitors (Laufer *et al.* 2004, Ziegler *et al.* 2009).

The title compound, *N*-{4-[4-(4-fluorophenyl)-1-(2-methoxyethyl)-2-(methylsulfanyl)-1*H*-imidazol-5-yl]pyridin-2-yl}-2-methyl-3-phenylpropionamide (I), was prepared in the course of our studies on 5-(2-acylpyridin-4-yl)-4-(4-fluorophenyl)-1-(2-methoxyethyl)-2-(methylsulfanyl)-1*H*-imidazoles as potent p38 MAP kinase inhibitors (Ziegler *et al.* 2009).

As might be expected the 4-fluorophenyl, the pyridine ring, the phenyl as well as the imidazole ring are planar (Figure 1). The imidazole ring makes dihedral angles of 11.85 (7)°, 73.33 (7)° and 22.83 (8)° to the 4-fluorophenyl ring (C30–C36), the pyridine ring and the phenyl ring (C13–C18), respectively. The 4-fluorophenyl ring (C30–C36) makes dihedral angles of 77.91 (7)° and 26.93 (8)° to the pyridine and the phenyl ring (C13–C18). The phenyl ring (C13—C18) and the pyridine ring are nearly perpendicular to one another with a dihedral angle of 86.47 (9)°. The amide function forms an N–H···O hydrogen bond to O9 of a symmetry related molecule resulting in an infinite chain of alternated enantiomers along the *c* axis of the unit cell (Figure 2). The length of the hydrogen bond is 2.21Å (Table 1).

### Experimental

Under an argon atmosphere, *N,N'*-carbonyldiimidazole (0.45 g, 2.8 mmol, 1 eq.) was added to a stirred solution of the 2-methyl-3-phenylpropionic acid (0.46 g, 2.8 mmol, 1.0 eq.) in 50 ml dry tetrahydrofuran at room temperature. When the effervescence stopped, 4-[5-(4-fluorophenyl)-3-(2-methoxyethyl)-2-methylsulfanyl-3*H*-imidazol-4-yl]-pyridin-2-ylamine (1.0 g, 2.8 mmol, 1 eq.) was added and the reaction was allowed to proceed at room temperature for three hours until the reaction was completed. After removing the solvent *in vacuo* ethylacetate was added to the residue. The organic layer was washed with water, dried over Na_2_SO_4_ and concentrated *in vacuo*. The crude product was purified *via* column chromatography (RP-18, acetonitrile: water = 6:4) yielding the title compound as a lightly brown solid (130 mg, 0.26 mmol, 9%). Crystals of compound I suitable for X-ray diffraction were obtained by slow evaporation at 298 K of a solution in n-hexane/diethyl ether.

### Refinement

Hydrogen atoms attached to carbons were placed at calculated positions with C—H = 0.95 Å (aromatic) or 0.98–0.99 Å (*sp*^3^ C-atom). The hydrogen atom attached to N7 was located from difference Fourier maps. All H atoms were refined using the riding-model approximation with isotropic displacement parameters (set at 1.2–1.5 times of the *U*_eq_ of the parent atom).

### Crystal data

**Table:**

C_28_H_29_FN_4_O_2_S	*F*(000) = 1064
*M**_r_* = 504.61	*D*_x_ = 1.272 Mg m^−^^3^
Monoclinic, *C**c*	Mo *K*α radiation, λ = 0.71073 Å
Hall symbol: C -2yc	Cell parameters from 9849 reflections
*a* = 10.6254 (5) Å	θ = 2.3–27.8°
*b* = 28.542 (1) Å	µ = 0.16 mm^−^^1^
*c* = 9.8380 (4) Å	*T* = 173 K
β = 117.953 (1)°	Plate, colourless
*V* = 2635.5 (2) Å^3^	0.50 × 0.50 × 0.20 mm
*Z* = 4	

### Data collection

**Table:**

Bruker SMART CCD diffractometer	6183 independent reflections
Radiation source: sealed Tube	6019 reflections with *I* > 2σ(*I*)
graphite	*R*_int_ = 0.020
CCD scan	θ_max_ = 27.8°, θ_min_ = 1.4°
Absorption correction: multi-scan (*SADABS*; Bruker, 2006)	*h* = −13→13
*T*_min_ = 0.685, *T*_max_ = 0.746	*k* = −36→36
21744 measured reflections	*l* = −12→12

### Refinement

**Table:**

Refinement on *F*^2^	Secondary atom site location: difference Fourier map
Least-squares matrix: full	Hydrogen site location: inferred from neighbouring sites
*R*[*F*^2^ > 2σ(*F*^2^)] = 0.029	H-atom parameters constrained
*wR*(*F*^2^) = 0.078	*w* = 1/[σ^2^(*F*_o_^2^) + (0.0517*P*)^2^ + 0.6111*P*] where *P* = (*F*_o_^2^ + 2*F*_c_^2^)/3
*S* = 1.03	(Δ/σ)_max_ = 0.003
6183 reflections	Δρ_max_ = 0.27 e Å^−^^3^
328 parameters	Δρ_min_ = −0.17 e Å^−^^3^
2 restraints	Absolute structure: Flack (1983), 3058 Friedel pairs
Primary atom site location: structure-invariant direct methods	Flack parameter: 0.13 (4)

### Special details

**Table:**

Geometry. All e.s.d.'s (except the e.s.d. in the dihedral angle between two l.s. planes) are estimated using the full covariance matrix. The cell e.s.d.'s are taken into account individually in the estimation of e.s.d.'s in distances, angles and torsion angles; correlations between e.s.d.'s in cell parameters are only used when they are defined by crystal symmetry. An approximate (isotropic) treatment of cell e.s.d.'s is used for estimating e.s.d.'s involving l.s. planes.
Refinement. Refinement of *F*^2^ against ALL reflections. The weighted *R*-factor *wR* and goodness of fit *S* are based on *F*^2^, conventional *R*-factors *R* are based on *F*, with *F* set to zero for negative *F*^2^. The threshold expression of *F*^2^ > σ(*F*^2^) is used only for calculating *R*-factors(gt) *etc*. and is not relevant to the choice of reflections for refinement. *R*-factors based on *F*^2^ are statistically about twice as large as those based on *F*, and *R*- factors based on ALL data will be even larger.

### Fractional atomic coordinates and isotropic or equivalent isotropic displacement parameters (Å^2^)

**Table:**

	*x*	*y*	*z*	*U*_iso_*/*U*_eq_	
C1	0.24375 (13)	0.39688 (4)	0.21997 (14)	0.0222 (2)	
H1	0.2260	0.4140	0.1298	0.027*	
C2	0.28100 (12)	0.34968 (4)	0.23423 (13)	0.0205 (2)	
C3	0.30589 (15)	0.32580 (5)	0.36839 (15)	0.0281 (3)	
H3	0.3316	0.2936	0.3816	0.034*	
C4	0.29180 (17)	0.35060 (5)	0.48168 (16)	0.0331 (3)	
H4	0.3079	0.3343	0.5726	0.040*	
N5	0.25706 (13)	0.39587 (4)	0.47118 (13)	0.0295 (2)	
C6	0.23312 (13)	0.41848 (4)	0.34154 (14)	0.0225 (2)	
N7	0.19830 (12)	0.46584 (4)	0.34381 (12)	0.0262 (2)	
H7	0.1880	0.4758	0.4166	0.031*	
C8	0.17895 (15)	0.49964 (4)	0.23856 (15)	0.0268 (3)	
O9	0.18466 (13)	0.49193 (3)	0.11861 (11)	0.0367 (2)	
C10	0.15915 (17)	0.54895 (5)	0.28535 (17)	0.0317 (3)	
H10	0.1283	0.5465	0.3667	0.038*	
C11	0.30411 (19)	0.57371 (6)	0.35365 (19)	0.0403 (3)	
H11A	0.2944	0.6056	0.3846	0.060*	
H11B	0.3375	0.5751	0.2763	0.060*	
H11C	0.3730	0.5563	0.4437	0.060*	
C12	0.04625 (17)	0.57601 (5)	0.14924 (18)	0.0349 (3)	
H12A	0.0524	0.6095	0.1780	0.042*	
H12B	0.0682	0.5737	0.0621	0.042*	
C13	−0.10448 (17)	0.55944 (5)	0.09573 (18)	0.0344 (3)	
C14	−0.1682 (2)	0.52638 (6)	−0.0202 (2)	0.0451 (4)	
H14	−0.1143	0.5122	−0.0635	0.054*	
C15	−0.3093 (2)	0.51393 (7)	−0.0734 (3)	0.0575 (5)	
H15	−0.3517	0.4915	−0.1537	0.069*	
C16	−0.3892 (2)	0.53379 (7)	−0.0109 (3)	0.0623 (6)	
H16	−0.4863	0.5253	−0.0484	0.075*	
C17	−0.3265 (2)	0.56607 (7)	0.1068 (3)	0.0559 (5)	
H17	−0.3806	0.5798	0.1506	0.067*	
C18	−0.1852 (2)	0.57843 (6)	0.1611 (2)	0.0424 (4)	
H18	−0.1424	0.6001	0.2437	0.051*	
C19	0.29361 (12)	0.32572 (4)	0.10753 (13)	0.0203 (2)	
N20	0.42522 (11)	0.31378 (4)	0.11967 (11)	0.0215 (2)	
C21	0.39870 (12)	0.29288 (4)	−0.01583 (13)	0.0215 (2)	
N22	0.26214 (11)	0.29049 (4)	−0.11212 (12)	0.0224 (2)	
C23	0.19440 (13)	0.31074 (4)	−0.03559 (13)	0.0203 (2)	
C24	0.56487 (13)	0.32057 (5)	0.25437 (14)	0.0254 (2)	
H24A	0.6281	0.2944	0.2598	0.031*	
H24B	0.5531	0.3194	0.3484	0.031*	
C25	0.63583 (15)	0.36626 (5)	0.25252 (17)	0.0320 (3)	
H25A	0.5692	0.3926	0.2341	0.038*	
H25B	0.7210	0.3713	0.3532	0.038*	
O26	0.67588 (14)	0.36466 (4)	0.13492 (14)	0.0423 (3)	
C27	0.7462 (4)	0.40628 (8)	0.1299 (4)	0.0772 (8)	
H27A	0.8367	0.4091	0.2247	0.116*	
H27B	0.6859	0.4334	0.1204	0.116*	
H27C	0.7645	0.4052	0.0412	0.116*	
S28	0.53568 (3)	0.271151 (12)	−0.04958 (4)	0.03048 (8)	
C29	0.43761 (19)	0.22789 (7)	−0.1920 (2)	0.0564 (6)	
H29A	0.3579	0.2429	−0.2797	0.085*	
H29B	0.4008	0.2042	−0.1475	0.085*	
H29C	0.5004	0.2128	−0.2267	0.085*	
C30	0.03784 (13)	0.31078 (4)	−0.11083 (14)	0.0210 (2)	
C31	−0.03604 (13)	0.29825 (4)	−0.26672 (14)	0.0244 (2)	
H31	0.0158	0.2916	−0.3212	0.029*	
C32	−0.18352 (14)	0.29538 (5)	−0.34257 (15)	0.0287 (3)	
H32	−0.2330	0.2862	−0.4476	0.034*	
C34	−0.25631 (14)	0.30601 (5)	−0.26286 (16)	0.0299 (3)	
C35	−0.18915 (14)	0.31932 (5)	−0.11006 (16)	0.0299 (3)	
H35	−0.2426	0.3268	−0.0580	0.036*	
C36	−0.04094 (14)	0.32145 (5)	−0.03438 (15)	0.0261 (3)	
H36	0.0073	0.3303	0.0710	0.031*	
F37	−0.40101 (9)	0.30368 (4)	−0.33851 (11)	0.0470 (2)	

### Atomic displacement parameters (Å^2^)

**Table:**

	*U*^11^	*U*^22^	*U*^33^	*U*^12^	*U*^13^	*U*^23^
C1	0.0269 (6)	0.0217 (5)	0.0189 (5)	0.0019 (4)	0.0115 (4)	0.0007 (4)
C2	0.0211 (5)	0.0213 (5)	0.0175 (5)	−0.0001 (4)	0.0075 (4)	−0.0016 (4)
C3	0.0393 (7)	0.0210 (6)	0.0253 (6)	0.0048 (5)	0.0163 (5)	0.0033 (5)
C4	0.0508 (8)	0.0283 (7)	0.0243 (6)	0.0066 (6)	0.0210 (6)	0.0072 (5)
N5	0.0423 (6)	0.0281 (6)	0.0232 (5)	0.0056 (5)	0.0197 (5)	0.0013 (4)
C6	0.0258 (5)	0.0211 (6)	0.0224 (5)	0.0016 (4)	0.0129 (4)	−0.0002 (4)
N7	0.0392 (6)	0.0230 (5)	0.0226 (5)	0.0063 (4)	0.0196 (5)	−0.0012 (4)
C8	0.0366 (7)	0.0211 (6)	0.0263 (6)	0.0057 (5)	0.0177 (5)	0.0006 (5)
O9	0.0664 (7)	0.0250 (5)	0.0277 (5)	0.0096 (4)	0.0296 (5)	0.0035 (4)
C10	0.0481 (8)	0.0214 (6)	0.0323 (7)	0.0063 (5)	0.0244 (6)	−0.0012 (5)
C11	0.0530 (9)	0.0291 (7)	0.0381 (8)	0.0007 (6)	0.0207 (7)	−0.0063 (6)
C12	0.0496 (8)	0.0253 (6)	0.0385 (7)	0.0095 (6)	0.0278 (7)	0.0060 (6)
C13	0.0475 (8)	0.0233 (6)	0.0365 (7)	0.0095 (6)	0.0230 (6)	0.0070 (5)
C14	0.0605 (10)	0.0306 (7)	0.0449 (9)	0.0085 (7)	0.0254 (8)	0.0024 (6)
C15	0.0654 (12)	0.0364 (9)	0.0530 (10)	−0.0024 (8)	0.0130 (9)	0.0001 (8)
C16	0.0491 (11)	0.0429 (10)	0.0877 (16)	0.0014 (8)	0.0262 (11)	0.0206 (10)
C17	0.0613 (11)	0.0424 (10)	0.0804 (14)	0.0098 (8)	0.0469 (11)	0.0124 (9)
C18	0.0545 (10)	0.0321 (8)	0.0483 (9)	0.0076 (7)	0.0304 (8)	0.0035 (6)
C19	0.0226 (5)	0.0178 (5)	0.0206 (5)	0.0018 (4)	0.0101 (4)	0.0013 (4)
N20	0.0221 (5)	0.0215 (5)	0.0203 (5)	0.0007 (4)	0.0093 (4)	−0.0010 (4)
C21	0.0233 (6)	0.0210 (5)	0.0219 (5)	−0.0022 (4)	0.0121 (5)	−0.0024 (4)
N22	0.0244 (5)	0.0223 (5)	0.0217 (5)	0.0000 (4)	0.0118 (4)	−0.0013 (4)
C23	0.0256 (6)	0.0163 (5)	0.0194 (5)	0.0005 (4)	0.0109 (5)	0.0002 (4)
C24	0.0222 (5)	0.0295 (6)	0.0202 (5)	0.0001 (4)	0.0062 (4)	−0.0018 (4)
C25	0.0317 (7)	0.0298 (7)	0.0331 (7)	−0.0058 (5)	0.0140 (6)	−0.0099 (5)
O26	0.0589 (7)	0.0308 (5)	0.0498 (6)	−0.0129 (5)	0.0359 (6)	−0.0098 (5)
C27	0.129 (2)	0.0429 (10)	0.1070 (19)	−0.0351 (13)	0.0947 (19)	−0.0215 (12)
S28	0.02474 (14)	0.04109 (18)	0.03028 (15)	−0.00436 (13)	0.01678 (12)	−0.00972 (14)
C29	0.0352 (8)	0.0700 (12)	0.0673 (12)	−0.0093 (8)	0.0267 (8)	−0.0471 (10)
C30	0.0235 (5)	0.0166 (5)	0.0212 (5)	0.0001 (4)	0.0090 (4)	0.0017 (4)
C31	0.0261 (6)	0.0222 (6)	0.0231 (6)	0.0036 (4)	0.0100 (5)	−0.0009 (4)
C32	0.0273 (6)	0.0277 (6)	0.0230 (6)	0.0003 (5)	0.0051 (5)	−0.0017 (5)
C34	0.0220 (6)	0.0307 (6)	0.0306 (7)	−0.0028 (5)	0.0069 (5)	0.0023 (5)
C35	0.0270 (6)	0.0364 (7)	0.0299 (7)	−0.0043 (5)	0.0165 (5)	−0.0004 (5)
C36	0.0265 (6)	0.0304 (7)	0.0208 (5)	−0.0042 (5)	0.0106 (5)	−0.0006 (4)
F37	0.0225 (4)	0.0728 (7)	0.0388 (5)	−0.0065 (4)	0.0086 (4)	−0.0028 (5)

### Geometric parameters (Å, °)

**Table:**

C1—C2	1.3926 (16)	C18—H18	0.9500
C1—C6	1.3963 (17)	C19—C23	1.3730 (16)
C1—H1	0.9500	C19—N20	1.3891 (15)
C2—C3	1.3962 (17)	N20—C21	1.3638 (15)
C2—C19	1.4818 (16)	N20—C24	1.4670 (15)
C3—C4	1.3865 (19)	C21—N22	1.3111 (16)
C3—H3	0.9500	C21—S28	1.7511 (12)
C4—N5	1.3344 (18)	N22—C23	1.3882 (15)
C4—H4	0.9500	C23—C30	1.4700 (16)
N5—C6	1.3429 (16)	C24—C25	1.5106 (19)
C6—N7	1.4047 (16)	C24—H24A	0.9900
N7—C8	1.3584 (17)	C24—H24B	0.9900
N7—H7	0.8242	C25—O26	1.4083 (18)
C8—O9	1.2295 (16)	C25—H25A	0.9900
C8—C10	1.5257 (17)	C25—H25B	0.9900
C10—C12	1.524 (2)	O26—C27	1.416 (2)
C10—C11	1.534 (2)	C27—H27A	0.9800
C10—H10	1.0000	C27—H27B	0.9800
C11—H11A	0.9800	C27—H27C	0.9800
C11—H11B	0.9800	S28—C29	1.7902 (16)
C11—H11C	0.9800	C29—H29A	0.9800
C12—C13	1.508 (2)	C29—H29B	0.9800
C12—H12A	0.9900	C29—H29C	0.9800
C12—H12B	0.9900	C30—C36	1.3962 (18)
C13—C14	1.388 (2)	C30—C31	1.4026 (16)
C13—C18	1.400 (2)	C31—C32	1.3867 (18)
C14—C15	1.383 (3)	C31—H31	0.9500
C14—H14	0.9500	C32—C34	1.369 (2)
C15—C16	1.382 (3)	C32—H32	0.9500
C15—H15	0.9500	C34—F37	1.3599 (15)
C16—C17	1.382 (3)	C34—C35	1.381 (2)
C16—H16	0.9500	C35—C36	1.3924 (18)
C17—C18	1.383 (3)	C35—H35	0.9500
C17—H17	0.9500	C36—H36	0.9500
			
C2—C1—C6	118.29 (11)	C23—C19—N20	105.65 (10)
C2—C1—H1	120.9	C23—C19—C2	132.66 (11)
C6—C1—H1	120.9	N20—C19—C2	121.69 (10)
C1—C2—C3	119.11 (11)	C21—N20—C19	106.55 (10)
C1—C2—C19	119.69 (10)	C21—N20—C24	126.79 (10)
C3—C2—C19	121.20 (11)	C19—N20—C24	126.61 (10)
C4—C3—C2	117.82 (11)	N22—C21—N20	112.37 (10)
C4—C3—H3	121.1	N22—C21—S28	125.54 (9)
C2—C3—H3	121.1	N20—C21—S28	122.07 (9)
N5—C4—C3	124.21 (12)	C21—N22—C23	105.42 (10)
N5—C4—H4	117.9	C19—C23—N22	109.99 (10)
C3—C4—H4	117.9	C19—C23—C30	131.29 (11)
C4—N5—C6	117.46 (11)	N22—C23—C30	118.63 (10)
N5—C6—C1	123.09 (11)	N20—C24—C25	113.73 (11)
N5—C6—N7	112.43 (11)	N20—C24—H24A	108.8
C1—C6—N7	124.48 (11)	C25—C24—H24A	108.8
C8—N7—C6	128.19 (11)	N20—C24—H24B	108.8
C8—N7—H7	112.5	C25—C24—H24B	108.8
C6—N7—H7	119.3	H24A—C24—H24B	107.7
O9—C8—N7	123.12 (11)	O26—C25—C24	109.06 (11)
O9—C8—C10	122.05 (12)	O26—C25—H25A	109.9
N7—C8—C10	114.71 (11)	C24—C25—H25A	109.9
C12—C10—C8	111.44 (12)	O26—C25—H25B	109.9
C12—C10—C11	111.51 (12)	C24—C25—H25B	109.9
C8—C10—C11	107.76 (12)	H25A—C25—H25B	108.3
C12—C10—H10	108.7	C25—O26—C27	111.64 (14)
C8—C10—H10	108.7	O26—C27—H27A	109.5
C11—C10—H10	108.7	O26—C27—H27B	109.5
C10—C11—H11A	109.5	H27A—C27—H27B	109.5
C10—C11—H11B	109.5	O26—C27—H27C	109.5
H11A—C11—H11B	109.5	H27A—C27—H27C	109.5
C10—C11—H11C	109.5	H27B—C27—H27C	109.5
H11A—C11—H11C	109.5	C21—S28—C29	99.33 (7)
H11B—C11—H11C	109.5	S28—C29—H29A	109.5
C13—C12—C10	114.68 (12)	S28—C29—H29B	109.5
C13—C12—H12A	108.6	H29A—C29—H29B	109.5
C10—C12—H12A	108.6	S28—C29—H29C	109.5
C13—C12—H12B	108.6	H29A—C29—H29C	109.5
C10—C12—H12B	108.6	H29B—C29—H29C	109.5
H12A—C12—H12B	107.6	C36—C30—C31	118.36 (11)
C14—C13—C18	118.23 (17)	C36—C30—C23	123.48 (11)
C14—C13—C12	121.94 (14)	C31—C30—C23	118.14 (11)
C18—C13—C12	119.81 (14)	C32—C31—C30	121.03 (12)
C15—C14—C13	120.69 (18)	C32—C31—H31	119.5
C15—C14—H14	119.7	C30—C31—H31	119.5
C13—C14—H14	119.7	C34—C32—C31	118.56 (12)
C16—C15—C14	120.62 (19)	C34—C32—H32	120.7
C16—C15—H15	119.7	C31—C32—H32	120.7
C14—C15—H15	119.7	F37—C34—C32	118.24 (12)
C17—C16—C15	119.40 (19)	F37—C34—C35	118.93 (13)
C17—C16—H16	120.3	C32—C34—C35	122.82 (12)
C15—C16—H16	120.3	C34—C35—C36	118.15 (12)
C16—C17—C18	120.22 (19)	C34—C35—H35	120.9
C16—C17—H17	119.9	C36—C35—H35	120.9
C18—C17—H17	119.9	C35—C36—C30	121.06 (12)
C17—C18—C13	120.78 (17)	C35—C36—H36	119.5
C17—C18—H18	119.6	C30—C36—H36	119.5
C13—C18—H18	119.6		
			
C6—C1—C2—C3	0.14 (18)	C2—C19—N20—C21	−179.52 (10)
C6—C1—C2—C19	−179.83 (11)	C23—C19—N20—C24	−177.12 (11)
C1—C2—C3—C4	0.12 (19)	C2—C19—N20—C24	2.80 (18)
C19—C2—C3—C4	−179.91 (12)	C19—N20—C21—N22	−0.15 (14)
C2—C3—C4—N5	−0.5 (2)	C24—N20—C21—N22	177.53 (11)
C3—C4—N5—C6	0.6 (2)	C19—N20—C21—S28	−178.85 (9)
C4—N5—C6—C1	−0.4 (2)	C24—N20—C21—S28	−1.17 (18)
C4—N5—C6—N7	−179.66 (13)	N20—C21—N22—C23	−0.33 (14)
C2—C1—C6—N5	−0.02 (19)	S28—C21—N22—C23	178.31 (9)
C2—C1—C6—N7	179.20 (11)	N20—C19—C23—N22	−0.79 (13)
N5—C6—N7—C8	174.22 (13)	C2—C19—C23—N22	179.31 (12)
C1—C6—N7—C8	−5.1 (2)	N20—C19—C23—C30	175.68 (12)
C6—N7—C8—O9	3.0 (2)	C2—C19—C23—C30	−4.2 (2)
C6—N7—C8—C10	−173.10 (13)	C21—N22—C23—C19	0.70 (13)
O9—C8—C10—C12	43.35 (19)	C21—N22—C23—C30	−176.27 (10)
N7—C8—C10—C12	−140.48 (13)	C21—N20—C24—C25	90.03 (15)
O9—C8—C10—C11	−79.29 (17)	C19—N20—C24—C25	−92.74 (15)
N7—C8—C10—C11	96.87 (14)	N20—C24—C25—O26	−68.96 (15)
C8—C10—C12—C13	71.17 (16)	C24—C25—O26—C27	−178.65 (19)
C11—C10—C12—C13	−168.37 (12)	N22—C21—S28—C29	−23.44 (14)
C10—C12—C13—C14	−92.71 (17)	N20—C21—S28—C29	155.08 (13)
C10—C12—C13—C18	88.86 (17)	C19—C23—C30—C36	−9.4 (2)
C18—C13—C14—C15	2.4 (2)	N22—C23—C30—C36	166.83 (11)
C12—C13—C14—C15	−176.06 (16)	C19—C23—C30—C31	172.30 (12)
C13—C14—C15—C16	−0.8 (3)	N22—C23—C30—C31	−11.49 (16)
C14—C15—C16—C17	−0.6 (3)	C36—C30—C31—C32	−1.56 (19)
C15—C16—C17—C18	0.2 (3)	C23—C30—C31—C32	176.85 (11)
C16—C17—C18—C13	1.5 (3)	C30—C31—C32—C34	1.4 (2)
C14—C13—C18—C17	−2.8 (2)	C31—C32—C34—F37	179.18 (13)
C12—C13—C18—C17	175.69 (15)	C31—C32—C34—C35	−0.2 (2)
C1—C2—C19—C23	−73.36 (17)	F37—C34—C35—C36	179.91 (13)
C3—C2—C19—C23	106.68 (16)	C32—C34—C35—C36	−0.7 (2)
C1—C2—C19—N20	106.75 (13)	C34—C35—C36—C30	0.5 (2)
C3—C2—C19—N20	−73.21 (16)	C31—C30—C36—C35	0.62 (19)
C23—C19—N20—C21	0.57 (12)	C23—C30—C36—C35	−177.70 (12)

### Hydrogen-bond geometry (Å, °)

**Table:**

*D*—H···*A*	*D*—H	H···*A*	*D*···*A*	*D*—H···*A*
N7—H7···O9^i^	0.82	2.21	3.025 (1)	173

Symmetry codes: (i) *x*, −*y*+1, *z*+1/2.
